# Supplementary material for: Splice-Junction-Based Mapping of Alternative Isoforms in the Human Proteome
Source: Cell Rep. Author manuscript; Available in PMC 2020 Jan 15. (PMC6961840; doi:10.1016/j.celrep.2019.11.026)
Supplement: 2 [file NIHMS1546469-supplement-2.zip › DF1/PXD006675/AorticValve/AorticValve_3_TTYH3_YLATSQPRPDSSGSAR.pdf]

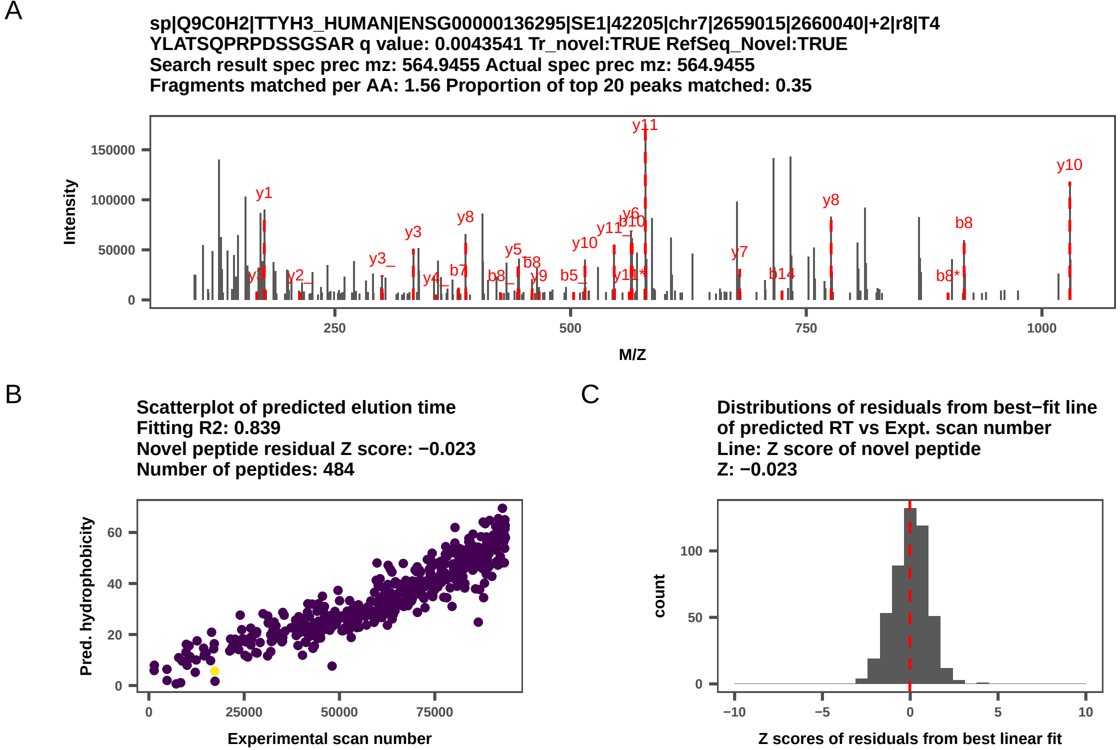
A
sp|Q9C0H2|TTYH3_HUMAN|ENSG00000136295|SE1|42205|chr7|2659015|2660040|+2|r8|T4
YLATSQPRPDSSGSAR q value: 0.0043541 Tr_novel:TRUE RefSeq_Novel:TRUE
Search result spec prec mz: 564.9455 Actual spec prec mz: 564.9455
Fragments matched per AA: 1.56 Proportion of top 20 peaks matched: 0.35
Intensity
150000
100000
50000
0
250
500
750
1000
M/Z
y11
y10
y1
y8
y3
y8
b8
y7
b14
b8*
y3_
y2_
y4_
b7
b8_
y5
b8
y9
b5_
y10
y11_
y6
b10
y11*
B
Scatterplot of predicted elution time
Fitting R2: 0.839
Novel peptide residual Z score: −0.023
Number of peptides: 484
Pred. hydrophobicity
60
40
20
0
0
25000
50000
75000
Experimental scan number
C
Distributions of residuals from best−fit line
of predicted RT vs Expt. scan number
Line: Z score of novel peptide
Z: −0.023
count
100
50
0
−10
−5
0
5
10
Z scores of residuals from best linear fit
